# Phytochemically Functionalized Cu and Ag Nanoparticles Embedded in MWCNTs for Enhanced Antimicrobial and Anticancer Properties

**DOI:** 10.1007/s40820-015-0066-0

**Published:** 2015-10-15

**Authors:** S. Yallappa, J. Manjanna, B. L. Dhananjaya, U. Vishwanatha, B. Ravishankar, H. Gururaj, P. Niranjana, B. S. Hungund

**Affiliations:** 1 Department of Industrial Chemistry, Kuvempu University, Shankaraghatta, Shimoga-Dist, 577 451 India; 2 Department of Chemistry, Rani Channamma University, Belagavi, 591 156 India; 3 Toxicology and Drug Discovery Centre for Emerging Technologies, Jain University, Ramanagara, 562 112 India; 4 SDM Centre for Research in Ayurveda and Allied Sciences, Udupi, 574 118 India; 5 Department of Electronics, Kuvempu University, Shankarghatta, 577 451 India; 6 Department of Biochemistry, Kuvempu University, Shankarghatta, 577 451 India; 7 Department of Biotechnology, B.V.B. College of Engineering & Technology, Hubli, 580 031 India

**Keywords:** Cu**-**MWCNTs, Ag**-**MWCNTs, *Terminalia arjuna* bark extract, Antimicrobial activity, Anticancer activity

## Abstract

Nanomedicine is an emerging field concerned with the use of precision engineered nanomaterials, which leads to the development of novel remedial and diagnostic modalities for human use. In this study, Cu(NO_3_)_2_ and AgNO_3_ precursors were reduced to copper nanoparticles (CuNPs) and silver nanoparticles (AgNPs) using *Terminalia arjuna* bark extracts under microwave irradiation in the presence of well-dispersed multi-walled carbon nanotubes (MWCNTs) in aqueous medium. The formation of CuNPs or AgNPs and their functionalization with MWCNTs via bioactive molecules of plant extract were evidenced from UV–Vis spectra, XRD, FTIR, FESEM, EDX, and TEM images. The phytochemically functionalized Cu-MWCNTs and Ag-MWCNTs nanomaterials showed enhanced biocide activity, and the inhibitory activity for bacteria was higher than that of fungus. Furthermore, these biohybrid nanomaterials are non-toxic to normal epithelial cells (Vero), whereas they are highly toxic for tested human cancer cells of MDA-MB-231, HeLa, SiHa, and Hep-G2. The cell viability was found to decrease with the increasing dose from 10 to 50 µg mL^−1^, as well as incubation time from 24 to 72 h. For instance, the cell viability was found to be ~91 % for normal Vero cells and ~76 % for cancer cells for lower dose of 10 µg mL^−1^.

## Introduction

Since ancient times, the Cu and Ag metal nanoparticles (MNPs) are known for potential bactericidal properties. Based on the exhaustive antimicrobial studies, these NPs are extensively used in wound healing ointments, bandage cloths, dental work, food packaging, catheters, coating on clinical equipments, etc. [1–3]. Recently, Cu and Ag NPs are explored for their biomedical applications such as drug delivery [4, 5], gene delivery [6–8], and cancer therapy [9]. In order to obtain an enhanced biological activity and also to meet certain specific requirements, the composite nanomaterials are in demand. In this regard, CNTs can provide the best platform to host and/or conjugate with MNPs in the presence of bioactive molecules [10]. The combination of CNTs and MNPs could be a successful integration of the properties of two components, which can lead to the new class of hybrid nanomaterials having novel features with diverse applications. Such a metallization of CNTs is known to increase the active sites as well as maintain superior performance and excellent intrinsic properties [11]. However, it is of interest to study functionalization of CNTs with MNPs such as Ag, Cu, Fe, Pd, etc. for diversified applications [12–15]. Among them, CNTs functionalized with Cu and Ag NPs have received considerable attention owing to their excellent biocompatibility and inherent antimicrobial properties. Therefore, many synthetic approaches including chemical or electrochemical reductions [16, 17], vapor deposition [18], γ–irradiation [19], etc. have been attempted for the functionalization of CNT with Cu and Ag NPs. Nevertheless, environmental benign and convenient route is still more desired for the fabrication of well-constructed hybrid nanomaterials.

It is well known that Au, Ag, and Cu nanoparticles can be easily prepared by using various plant extracts. The bioactive components in the plant extracts consist of phytochemicals such as polyphenols, flavonoids, and reducing sugars, which are responsible for the reduction and capping process of MNPs. Recently, we have used *Terminalia arjuna* (*T. arjuna*) bark, an ethno medicinally important evergreen tree, for synthesis of bio-capped Cu and Ag NPs [20, 21]. The bio-reduced Cu and Ag NPs showed enhanced antimicrobial and antioxidant activity owing to bioactive components on these materials. In this study, we have followed a similar strategy to incorporate and/or embedding the Cu and Ag NPs in MWCNTs matrix. During this process, the conjugation of Cu and Ag NPs with MWCNTs occurs via bioactive molecules from plant extract. In our pursuit for materials with enhanced antimicrobial and anticancer activity, we found that these biohybrid materials are worth exploring. There are few reports on the antimicrobial studies of similar materials obtained by chemical route, for instance, Cu/Ag-MWCNTs [22] and Ag-MWCNTs [23]. To our knowledge, there are no studies on anticancer activity, especially with biohybrid materials. Thus, in addition to antimicrobial property, we have found enhanced anticancer activity against human cancer cell lines namely MDA-MB-231 (breast), HeLa (cervix), SiHa (cervix), and Hep-G2 (liver) for these synthesized biohybrid materials.

## Experimental

### Materials

The fresh bark extract of *T. arjuna* plant (collected from the Western Ghats of Karnataka state in India) was used here for both reduction of metal ions and their functionalization with MWCNTs (supplied from Adnano Technologies Private Ltd., Bangalore, India). MWCNTs were pretreated with nitric acid for few hours to remove any metal ions and also to impart functional groups such as –COOH to improve their dispersing ability in aqueous medium. Silver nitrate (AgNO_3_), copper nitrate (CuNO_3_)_2_, 3-(4,5-dimethyl-2-thiazolyl)-2, 5-diphenyl-2H-tetrazolium bromide (MTT), fetal calf serum (FCS), modified Eagles Medium (MEM), glutamine, EDTA, trypsin, dimethyl sulfoxide (DMSO), methanol, ciprofloxacin, fluconazole, and McFarland solution of analytical grade were purchased from Hi-media Laboratory, Bangalore, India. The bacterial strains of *Escherichia coli* (*E. coli*), *Staphylococcus aureus (S. aureus), Salmonella typhi (S. typhi)*, and *Pseudomonas aeruginosa* (*P. aeruginosa*) were obtained from Microbial Type Culture Collection (MTCC) at Chandigarh in India. The fungus strains of *Trichopyton rubrum* (*T.* *rubrum*), *Candida albicans* (*C. albicans*), and *Chrysosporium indicum* (*C. indicum*) were obtained from Green Gold Global Research Institute at Kumta in India and maintained in potato dextrose agar (Hi-Media, Bangalore, India) slant at 27 °C. The different cell lines viz., MDA-MB-231, HeLa, SiHa and Hep-G2 (cancer cells), and Vero (normal epithelial cells) were procured from National Centre for Cell Science (NCCS), India.

### Embedded or Biohybrid Structure of Cu-MWCNTs and Ag-MWCNTs

About 0.1 g of pretreated MWCNTs were dispersed in 50 mL of 10^−3^ M Cu(NO_3_)_2_ or AgNO_3_ solution and stirred for about 1 h at room temperature (RT). A freshly prepared 20 mL of *T. arjuna* bark extract, as reported in our previous study [20], was added to this suspension under continuous stirring. It was subjected for microwave irradiation (700 W, 2.45 GHz) for about 300 s. In the case of Cu-MWCNTs formation, the solution turned from light yellow to black in color. Similarly, Ag-MWCNTs formation was indicated by change in color from light yellow to dark brown. The formation of these biohybrid nanomaterials was monitored by UV–Vis spectrophotometer (Shimadzu, 1650-PC) for surface plasmon resonance (SPR) peak. To understand the reducing ability of the plant extract, the solution reduction potential (*E*) and pH of the reaction mixture were measured using digital potentiometer and pH meter, respectively. Finally, the nanobiohybrid solid mass thus obtained was washed thoroughly with distilled water and dried in vacuum oven at 70 °C for about 12 h to obtain the product in powder form. It was well characterized by using various physico-chemical techniques as given below.

### Characterization of Cu-MWCNTs and Ag-MWCNTs

The powder X-ray diffraction (XRD, Siemens X-ray diffractometer, Japan) pattern of MWCNTs, Cu-MWCNTs, and Ag-MWCNTs were recorded using ‘X’PERT-PRO XRPD (Cu Kα, *λ* = 0.15406 nm) with a scanning rate of 2° min^−1^ and 2*θ* ranging from 10° to 70°. The morphology of these prepared nanomaterials was observed by using field-emission scanning electron microscopy (FE-SEM, FEI Nova nano 600, Netherlands). The energy-dispersive X-ray (EDX) analysis was used for elemental composition. Transmission electron micrographs were obtained using TEM (JEOL, 200 kV), and the Fourier transform infrared spectra (FTIR, Bruker- TENSOR 27) were recorded in KBr pellet.

### In Vitro Antimicrobial Activity

The antimicrobial activity of all the samples (MWCNTs, Cu-MWCNTs and Ag-WCNTs) was tested under disk diffusion method on Muller Hinton agar (MHA) plates [24]. The standard antibiotic disks were procured from Hi-media Laboratory, Bangalore, India. To determine the antimicrobial activity, each std. antibiotic disks were further soaked or dipped with different concentrations of nanomaterials in DMSO viz., 100 % (10 µg mL^−1^), 75 % (7.5 µg mL^−1^), and 50 % (5.0 µg mL^−1^) and then dried in an oven at 30–40 °C. The microbial strains of bacteria and fungus were inoculated into the nutrient broth and kept on rotary shaker at 200 rpm for 24 h incubation at 37 °C. The turbidity of broth cultures was compared with that of std. 0.5 McFarland solutions and was applied to the MHA plates along with std. antibiotic disk and prepared disk containing differing amount of test samples. After incubation at 37 °C for 24 h, the zones of inhibition were measured using digimatic calipers (Mitutoyo Rochester, New York). The assays were performed in triplicate, and mean values of zone diameter were taken.

### In Vitro Anticancer Activity

#### Cell Culture

The normal cells (Vero) and human cancer cells (MDA-MB-231, HeLa, SiHa, and Hep-G2) were maintained in Modified Eagles Medium (MEM) supplemented with 10 % FCS, 2 % essential amino acids, 1 % each of glutamine, non-essential amino acids, vitamins, and 100 U/mL Penicillin–Streptomycin. Cells were sub-cultured at 80–90 % confluence and incubated at 37 °C in a humidified incubator supplied with 5 % CO_2_. The stock cells were maintained in 75 cm^2^ tissue culture flask.

#### Cell Viability Assay

The cytotoxicity effect of test samples was performed by 5-diphenyl-2H-tetrazolium bromide (MTT) assay [25]. Briefly, cultured cells (1 × 10^−6^ cells mL^−1^) were placed in 96 flat-bottom well plates, and then cells were exposed to different concentrations of prepared nanomaterials (1–100 µg mL^−1^) and incubated at 37 °C for about 24 h in 5 % CO_2_ atmosphere. After 24 h incubation, MTT (10 µL) was added to the incubated cancer cells. Then MTT-added cells were further incubated at 37 °C for about 4 h in 5 % CO_2_ atmosphere. Thereafter, the formazan crystals were dissolved in 200 µL of DMSO, and the absorbance was monitored in a colorimetric at 578 nm with reference filter as 630 nm. The cytotoxicity effect was calculated as

$${\text{Cytotoxicity }}\left( \% \right) = 1- 100 \times {{\left( {\text{Mean absorbance of toxicant}} \right)} \mathord{\left/ {\vphantom {{\left( {\text{Mean absorbance of toxicant}} \right)} {\left( {{\text{Mean absorbance of }}{-}{\text{ve control}}} \right)}}} \right. \kern-0pt} {\left( {{\text{Mean absorbance of }}{-}{\text{ve control}}} \right)}}$$

$${\text{Cell viability }}\left( \% \right) = 100 \, - {\text{ Cytotoxicity }}\left( \% \right)$$

#### Statistical Analysis

The statistical analysis values for all the experiments were expressed as ±SD. The data were performed using Student *t* test, where statistical significance was calculated for treated samples and untreated (as control) cells.

## Results and Discussion

**Fig. 1:**
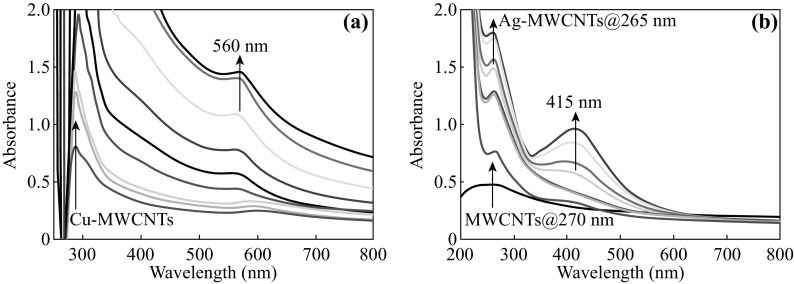
Optical absorption spectra of Cu-MWCNTs (**a**) and Ag-MWCNTs (**b**). *Inset* photos show the *color* of reaction mixture before and after 300 s of irradiation. (Color figure online)

As predicted by the well-known Mie resonance condition [26], CuNPs and AgNPs shows a characteristic SPR peak by absorbing light depending on the Bohr-radius and dielectric constant of the surrounding medium. As shown in Fig. 1, the UV–Vis spectra show *λ*_SPR_ for Cu-MWCNTs (Fig. 1a) ranging from 580 to 560 nm. Such a blue shift is expected for growing size of CuNPs embedded in the MWCNTs. Similarly, *λ*_SPR_ for Ag-MWCNTs (Fig. 1b) varied from 400 to 415 nm, exhibiting a red shift as expected for growing size of AgNPs embedded in the MWCNTs. The *λ*_SPR_ obtained here are in good agreement with those reported *λ*_SPR_ of Cu^0^ and Ag^0^ NPs [20, 21]. For comparison, MWCNTs dispersion alone showed *λ*_SPR_ ~270 nm, and it remained same but the intensity increased with microwave irradiation time. Inset photos show the color of reaction mixture before and after 300 s of microwave irradiation.

**Fig. 2:**
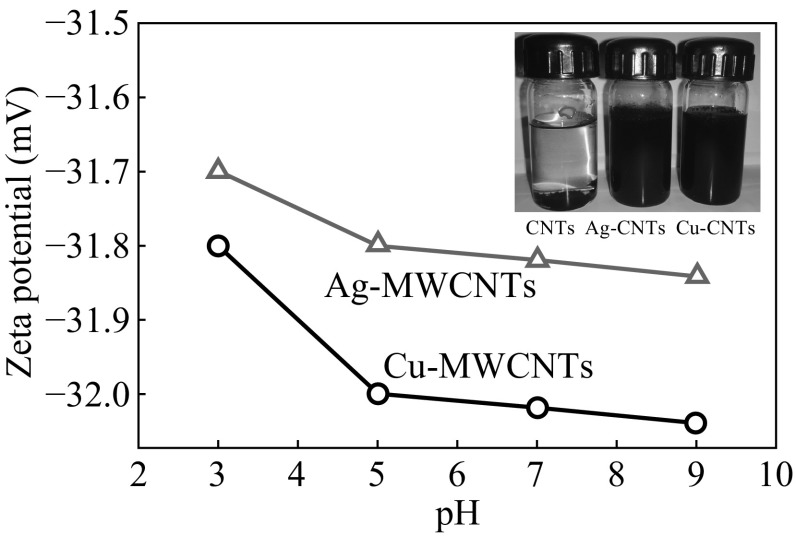
Zeta potential of the Cu-MWCNTs and Ag-MWCNTs dispersion at different pH. The *inset* shows the corresponding photos in comparison with pristine MWCNTs. (Color figure online)

The stability of nanomaterial is very important for exploring their biomedical applications [27]. The nanomaterials are typically stabilized by adding capping/stabilizing agents such as surfactants and polymers under various conditions. In this study, although we have not used any such external capping/stabilizing agents, considerable stability has been achieved. For instance, zeta potential is related to the chemical stability of colloidal dispersion, which indicates the degree of repulsion between adjacent, similarly charged particles in the dispersion media. Figure 2 shows the zeta potential of the reaction mixture at the end of reaction, i.e., Cu-MWCNTs and Ag-MWCNTs dispersions at different pH values. As shown in the inset photos, unlike the MWCNTs, the hybrid structure, i.e., Cu-MWCNTs and Ag-MWCNTs are well dispersed. Zeta potential decreased gradually, i.e., from ~31.8 to ~32.2 mV for Cu-MWCNTs and from ~31.7 to ~31.8 mV for Ag-MWCNTs over a pH range of 3–9. This is an indication of a stable dispersion, especially in the physiological pH range used here. The particles with zeta potential values ranging from +30 to −30 mV are normally considered as stable. Recently, Castillo et al. [27] have also observed a similar behavior of zeta potential for CNTs conjugated with folic acid. The stability in our samples may be due to bio-capping of phytoconstituents from plant extract. Such in situ bio-capped phytochemicals such as polyphenols, flavonoids, and reducing sugars can impart bio-conjugation between CuNPs or AgNPs and MWCNTs. This aspect has been clarified in the subsequent characterization using FTIR and FESEM/EDX analysis. The adsorption of plant bioactive molecules on these nanomaterials can be ascribed to the attractive force between positively charged surface of nanomaterials and the negatively charged bioactive molecules [28]. Such a bio-capping helps in preventing the agglomeration of NPs due to the repulsion among the bio-capped nanohybrids.

**Fig. 3:**
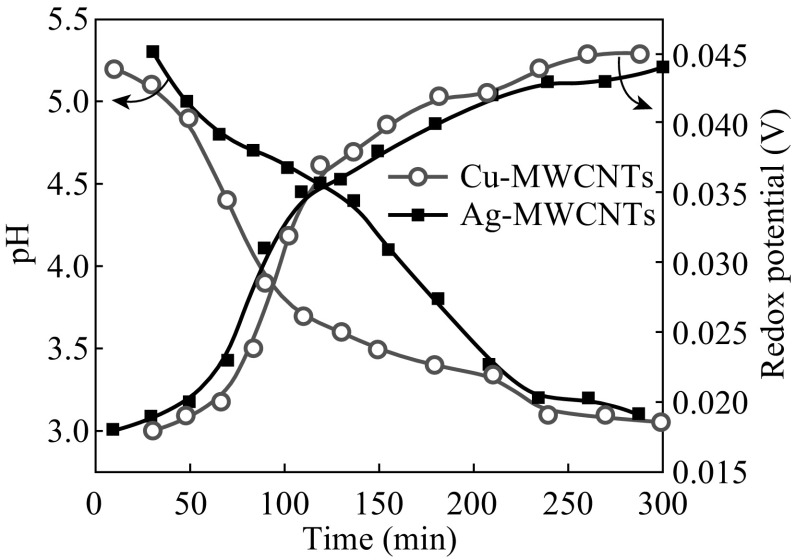
Variation of redox potential and pH of the reaction mixture as a function of irradiation time. (Color figure online)

To know the reducing ability of *T. arjuna* bark extract in the suspension of Cu^2+^/Ag^+^ ions dispersed with MWCNTs, the reduction potential (*E*) and pH of the reaction mixture were measured here. This procedure is useful for choosing a suitable plant material in metal ion reduction instead of random selection. The pH and *E* of the reaction mixture (Fig. 3) indicates reduction of Cu^2+^ to Cu^0^ and Ag^+^ to Ag^0^ particles on MWCNTs. The initial *E* = 0.018 V increased to 0.044 V after 300 s of microwave irradiation for Cu-MWCNTs, while *E* for Ag-MWCNTs was increased from 0.018 to 0.045 V. An increase in *E* to more positive values, although marginal, indicates the consumption of bioactive molecules from plant extract for rapid reduction of metal ions and their biofunctionalization with MWCNTs. In corroboration with these results, the initial pH 5.3 of the reaction mixture decreased to 3.1 for Cu-MWCNTs, whereas for Ag-MWCNTs, the pH was reduced from 5.2 to 3.05. This is due to the release of H^+^ ions during the oxidation of reductants present in the plant extract. A similar behavior was observed in our previous reports on CuNPs [20] and AgNPs [21] obtained by *T. Arjuna* bark extract. It is clear from *E* and pH of the reaction mixtures that *T. Arjuna* bark extract is highly compatible for the synthesis and biofunctionalization of MNPs with MWCNTs.

**Fig. 4:**
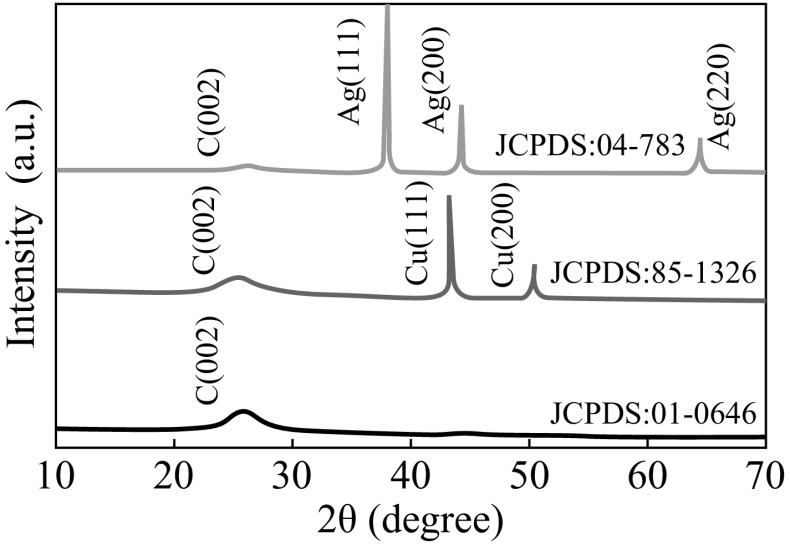
XRD pattern of pretreated MWCNTs, Cu-MWCNTs, and Ag-MWCNTs. (Color figure online)

The powder XRD pattern of MWCNTs, Cu-MWCNTs, and Ag-MWCNTs obtained here are shown in Fig. 4. The prominent diffraction peaks at 2*θ* = 26.24° are assigned to the (002) plane of the MWCNTs (JCPDS No. 01-0646) [29]. After embedded with CuNPs, the new peaks appeared at 2*θ* = 43.3° and 50.4° which can be indexed to (111) and (200) of *fcc*-structured CuNPs (JCPDS No. 85–1326), while the characteristic peak of MWCNTs remains unchanged. Similarly, in the case of Ag-MWCNTs, 2*θ* = 38.9°, 44.3°, and 64.49° peaks corresponding to (111), (200), and (220) of *fcc*-structured AgNPs (JCPDS No. 04–783) appeared. This is a clear indication for the formation of Cu-MWCNTs and Ag-MWCNTs heterostructures via functionalization and/or embedded process. The crystallite size *d* of these functionalized Cu and AgNPs was calculated to be ~20–30 nm using Scherer formula: *d* = *Kλ* **/** *β* cos*θ*, where *K* is the shape factor between 0.9 and 1.1, *λ* is the incident X-ray wavelength (Cu *K*α = 1.542 Å), *β* is the full width half-maximum in radians of the prominent line and *θ* is the position of that line in the pattern.

**Fig. 5:**
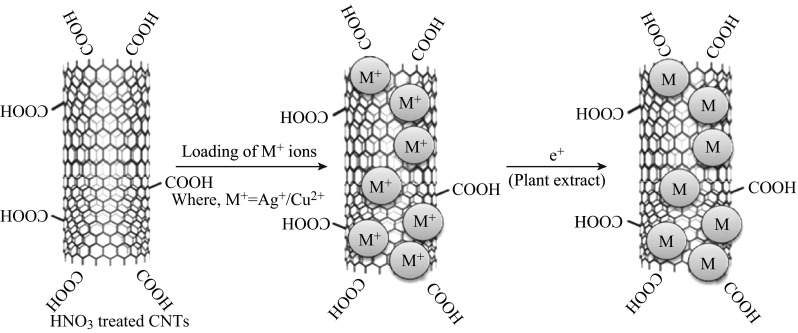
Schematic representation of Cu and Ag NPs embedded in MWCNTs. (Color figure online)

**Fig. 6:**
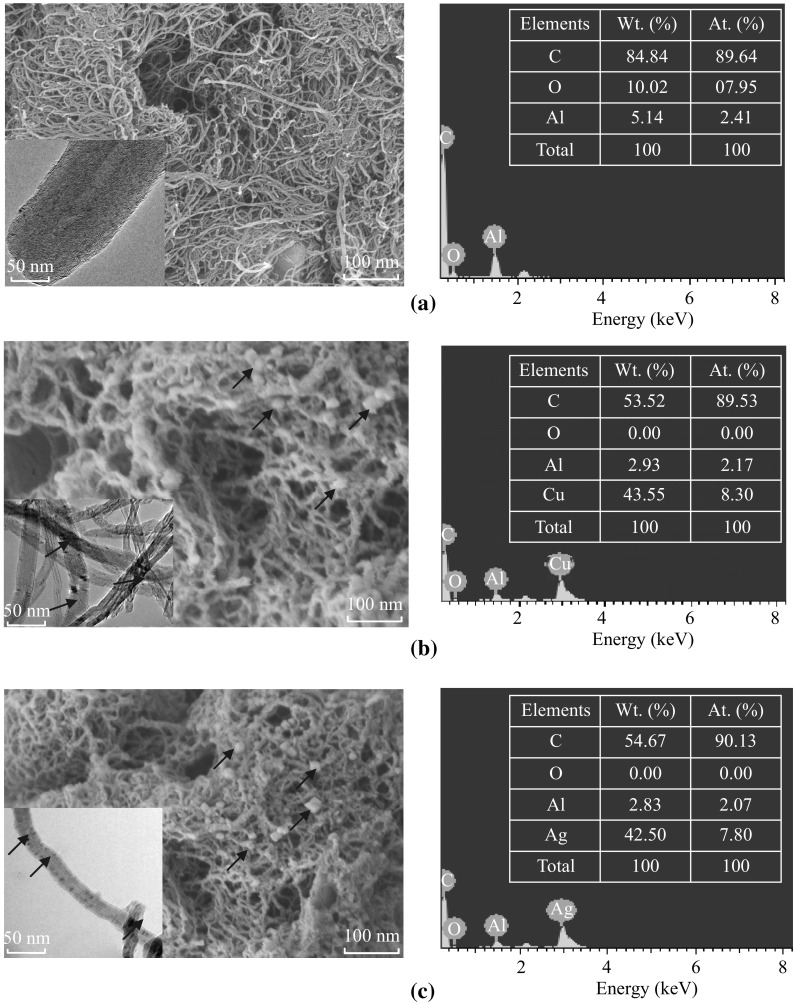
FESEM images and corresponding EDX spectra of MWCNTs (**a**), Cu-MWCNTs (**b**), and Ag-MWCNTs (**c**). The corresponding TEM images (*inset*) shows the smaller MNPs trapped (embedded) endohedrally along the inner surface of MWCNTs, while the exohedrally functionalized MNPs were removed when dispersed in ethanol and acetone mixture in ultrasonic bath. (Color figure online)

Figure 5 shows the schematic representation of Cu and AgNPs embedded in MWCNTs. Before reduction, the metal ions (the ionic radii of Cu^2+^: 0.73 Å and Ag^+^: 1.26 Å) are present at both endohedral and exohedral surfaces of MWCNTs. These metal ions are in situ reduced on microwave irradiation with the reducing agent, plant extract. Therefore, the Cu and AgNPs are embedded and/or functionalized both on endohedral and exohedral surfaces of MWCNTs. It is evident from their microstructural analysis. Figure 6 shows FESEM and EDX spectra of MWCNTs, Cu-MWCNTs, and Ag-MWCNTs. FESEM images clearly show somewhat wider distribution of particles ranging from few to 30 nm. In fact, these samples were carbon coated for providing conducting surface to minimize the charging effect while recording SEM images. On careful observation, we can see some kind of scum wrapped on these nano-hybrid structures (which is confirmed from FTIR spectra, as given below). There is not much difference in morphology of Cu-MWCNTs and Ag-MWCNTs. Such an amorphous part is due to bio-capping of medicinally important phytoconstituents (biomass), which also helps to protect nanomaterials from agglomeration as well as enhanced the antimicrobial and antiproliferative activity as reported here. The MNPs present here in the embedded form seemed to be more stable when compared to their formation without such platform i.e., MWCNTs. Although these heterostructures can be explored for their electronic behavior, we are interested in their biomedical applications. As expected, in EDX spectra (Fig. 6), only C, Cu, and Ag are seen as major elements. The calculated atomic ratio of C (MWCNTs) and Cu or Ag NPs is consistent with the intake ratio of 1:1. For TEM analysis (inset of Fig. 6b, c), the samples were dispersed in the ethanol/acetone mixture in ultrasonic bath, and a drop of supernatant solution was used for TEM sampling. During this treatment, the bio-capped MNPs are removed as the phytochemicals are soluble in these solvents; thereby, we do not see exohedrally functionalized nanoparticles, i.e., MNPs bound to the surface of MWCNTs are detached. However, the smaller MNPs trapped (embedded) endohedrally along the inner surface of MWCNTS could be clearly seen in TEM images. Thus, we see some discrepancy in the particle size as determined from XRD when compared to FESEM and TEM images.

**Fig. 7:**
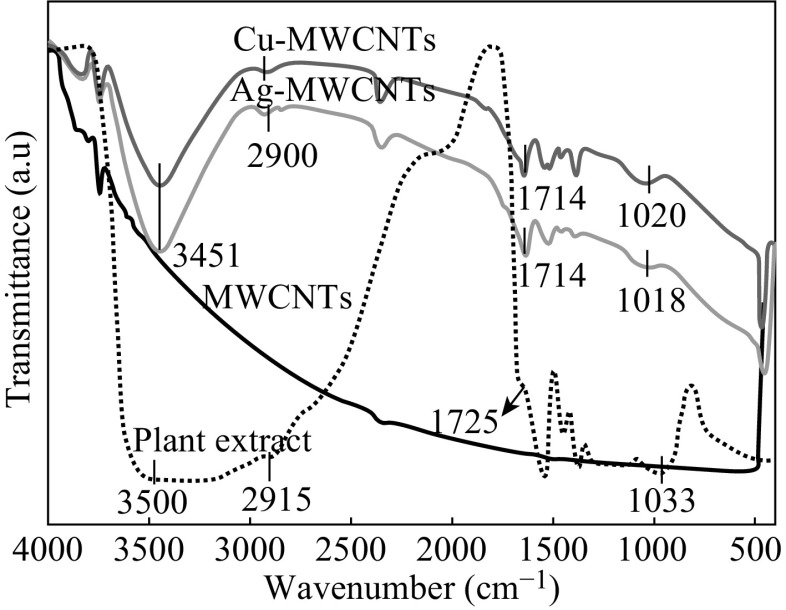
FTIR spectra of MWCNTs, Bio-functionalized Cu-MWCNTs, and Ag-MWCNTs. (Color figure online)

FTIR spectra (Fig. 7) were recorded to identify the bioactive molecules from *T. arjuna* plant extract responsible for the reduction of metal ions on MWCNTs. FTIR spectra of crude *T. arjuna* bark extract is are compared with MWCNTs, Cu-MWCNTs, and Ag-MWCNTs nanomaterials. In case of crude plant extract, the broad peaks around 3500 and 2915 cm^−1^ indicate the presence of O–H stretching of polyphenols. The bands at 1725 and 1033 cm^−1^ are attributed to C=O stretching and C–O bending frequencies [30]. These bands clearly indicate the presence of major bioactive molecules such as polyphenols, favonones, and terpenoids from plant extract. For Cu-MWCNTs and Ag-MWCNTs, there is a shift of IR bands viz., 2915–2900 cm^−1^, 1725–1714 cm^−1^, and 1034–1020 cm^−1^. Thus, some proteins and metabolites such as flavonoids, polyphenols, and terpenoids are involved in the reduction process of MNPs embedded on MWCNTs and bio-capping of these nanomaterials, thus by increasing their stability [30, 31]. Earlier reports have also confirmed the role of polyphenols and flavonoids in the reduction process of Cu^2+^ using *T. arjuna* bark [21] and Ag^+^ using *A. heterophyllus* leaves [32].

The Cu and AgNPs and their derivatives are well known for their broad-spectrum biocidal activity against bacterial and fungal agents without any cross resistance with antibiotics [33]. Thus, they are used for the treatment of burns and the chronic wounds [34]. The CNTs have also been found to possess antimicrobial property, although MWCNTs are known to be inferior when compared to SWCNTs towards bacteria [35]. It is advantageous to make use of MWCNTs having high surface area as substrates during the preparation of MNPs so that the MNPs will be embedded on MWCNTs resulting in biohybrid system, which is expected to show enhanced antimicrobial activity. The pretreated (acid digested) MWCNTs consists of carboxyl groups in the backbone that can bind with Cu^2+^ and Ag^+^ ions very easily, especially with these bio-capped MNPs. Tables 1, 2 show the antimicrobial activity of MWCNTs, Cu-MWCNTs, and Ag-MWCNTs on different bacteria and fungus under disk diffusion method. In general, Cu-MWCNTs and Ag-MWCNTs show enhanced inhibitory effect when compared to MWCNTs. The inhibitory activity for bacteria was found to be higher (*E. coli* > *S. typhi* > *S. aureus* > *P. aeruginosa*) than that of fungus (*C. albicans* > *T. rubrum* *≈* *C. indicum*). The enhanced antimicrobial activity of these nanobiohybrids can be attributed to the combined effect of CNTs, MNPs, and phytoconstituents from plant extract. These results suggest that the biohybrid nanomaterials can compete with commercial antimicrobial agents. Enhanced biocidal activity is reported [23, 36, 37] for composites of AgNPs-functionalized MWCNTs and AgNPs-functionalized trimolybdate nanowires when compared to MWCNTs, trimolybdate nanowires, and AgNPs alone.

**Table 1:** Antibacterial activity of MWCNT, Cu-MWCNTs, and Ag-MWCNTs

Microorganisms	Zone of inhibition (mm)				
	100 %	75 %	50 %	Ciprofloxacin (std.)	Control 10 % DMSO
(a) MWCNTs					
*S. aureus*	04	03	02	16	–
*S. typhi*	03	02	02	21	–
*E. coli*	04	03	02	20	–
*P. aeruginosa*	02	01	01	17	–
(b) Cu-MWCNTs					
*S. aureus*	14	13	10	16	–
*S. typhi*	15	12	11	21	–
*E. coli*	17	15	14	20	–
*P. aeruginosa*	10	08	08	17	–
(c) Ag-MWCNTs					
*S. aureus*	13	11	10	16	–
*S. typhi*	15	14	13	21	–
*E. coli*	16	13	11	20	–
*P. aeruginosa*	10	09	08	17	–

All experiments were performed in triplicate, and standard deviations were negligible

**Table 2:** Antifungal activity of MWCNT, Cu-MWCNTs, and Ag-MWCNTs

Microorganisms	Zone of inhibition (mm)				
	100 %	75 %	50 %	Fluconazole (std.)	Control 10 % DMSO
(a) MWCNTs					
*C. albicans*	03	03	02	13	–
*T. rubrum*	04	02	02	11	–
*C. indicum*	03	03	02	09	–
(b) Cu-MWCNTs					
*C. albicans*	08	06	06	13	–
*T. rubrum*	07	05	04	11	–
*C. indicum*	07	05	04	09	–
(c) Ag-MWCNTs					
*C. albicans*	08	07	06	13	–
*T. rubrum*	07	06	05	11	–
*C. indicum*	07	05	05	09	–

All experiments were performed in triplicate, and standard deviations were negligible

The precise mechanisms of bacterial growth inhibition by CuNPs and AgNPs have been extensively studied. For instance, Sondi and Salopek-Sondi [38] have proposed the formation of ‘pits’ in the cell wall, leading to cell death due to increased membrane permeability of *E. coli* cells when treated with AgNPs. For CuNPs also, a similar mechanism is speculated here. Since Cu and AgNPs are very reactive and can easily bind to tissue proteins, this induces structural changes in the bacterial cell wall and nuclear membrane that leads to cell death.

The antimicrobial activity observed here are consistent with a recent study by Rangari et al. [39] on Ag-CNT@polymer hybrid nanomaterial, which showed significant activity against bacterial strains, *E. coli* and *S. aureus*. Mohan et al. [22] have also reported that the surface area of MNPs was enhanced by embedding in CNTs; thereby, higher antimicrobial activity was observed. Hence, we have focused on new biohybrid materials, Cu-MWCNTs and Ag-MWCNTs. Indeed these nanomaterials show enhanced inhibitory activity as expected. To our knowledge, this is the first report of Cu-MWCNTs and Ag-MWCNTs using bio-inspired *T. arjuna* bark extract as reducing and/or biofunctionalizing agent under microwave irradiation. More investigations are required to determine the extent of their antimicrobial activity.

**Fig. 8:**
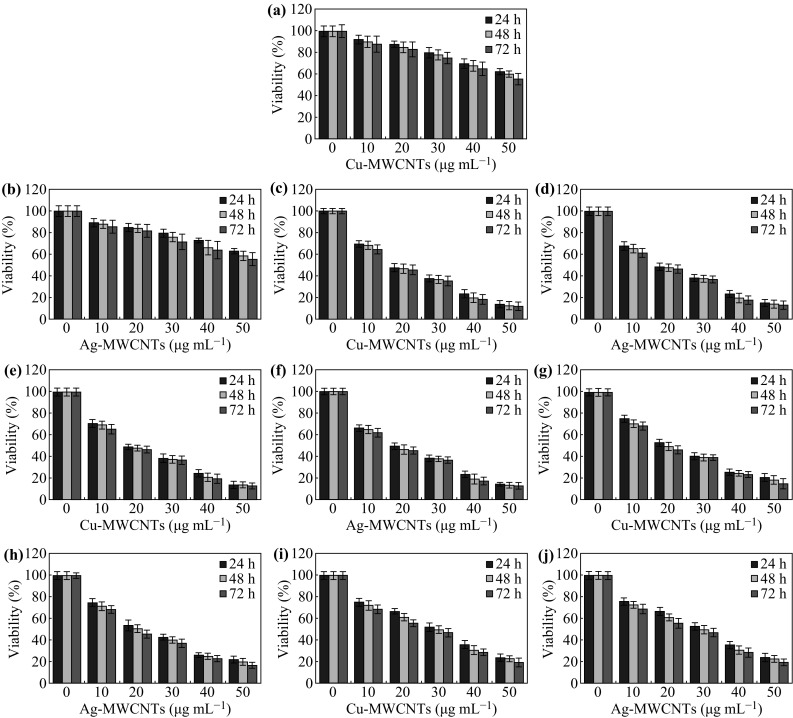
Cell viability assay of Vero (**a**, **b**), MDA-MB-231 (**c**, **d**), HeLa (**e**, **f**), SiHa (**g**, **h**), and Hep-G2 (**i**, **j**) after exposure to different concentrations of Cu-MWCNTs and Ag-MWCNTs nanobiohybrids at 24, 48, and 72 h. The values represent the mean ± SD of three independent experiments. (Color figure online)

We have also evaluated the cytotoxic effect of biohybrid nanomaterials (Cu-MWCNTs and Ag-MWCNTs) on different cell lines viz., MDA-MB-231, HeLa, SiHa and Hep-G2 (cancer cells), and Vero (normal epithelial cells). Figure 8 shows the effect of Cu-MWCNTs and Ag-MWCNTs on normal and cancer cells after incubating for 24–72 h with different dose levels, 10–50 µg mL^−1^. The viability of cells was found to decrease with increasing dose, 10–50 µg mL^−1^ as well as incubation time, 24–72 h. The cell viability of Cu-MWCNTs and Ag-MWCNTs at lower dose (10 µg mL^−1^) was found to be ~91 % for normal cells and ~76 % for cancer cells. However, there was a gradual decrease in the viability of cells with the increasing dose of Cu-MWCNTs and Ag-MWCNTs. At higher dose of 50 µg mL^−1^, the cell viability of Vero, MDA-MB-231, HeLa, SiHa, and Hep-G2 cells were dropped to ~60, 12, 15, 13, and 20 %, respectively, after 48 h exposure. The IC_50_ values and  % inhibition of cell proliferation of these biohybrid nanomaterials are shown in Table 3. This effective cytotoxicity of hetero-structured Cu-MWCNTs and Ag-MWCNTs on human cancer cells is attributed to the combined effect of MWCNTs embedded with MNPs as well as bio-capped organic moieties (phytochemicals) from plant extract to make the biohybrid nanomaterial. On the other hand, normal epithelial Vero cells showed excellent biocompatibility for Cu-MWCNTs and Ag-MWCNTs. This could be due to the presence of cell growth boosting factors in the *T. arjuna* bark extract and the high content of bioactive molecules such as polyphenols, terpenoids, reducing sugars, and other hydrophobic organic moieties which enable easy internalization of nanomaterials via hydrophobic interior of the cell membrane [40]. Recently, Molina et al. [41] have reported that inorganic nanomaterials profoundly interact with cells and intracellular macromolecules like proteins and DNA. Cellular uptake of NPs leads to generation of reactive oxygen species which provoke the oxidative stress. The Cu-MWCNTs and Ag-MWCNTs here might induce cell damage through the loss of cell membrane. However, several factors such as dose, exposure time, and size of the nanomaterials can influence the suppression of cancer cells growth. We envisage that the biohybrids nanomaterials obtained here could be used as potential therapeutic carriers for detection and destruction of cancer cells.

**Table 3:** Growth-inhibitory effect (after 48 h) of Cu-MWCNTs and Ag-MWCNTs on different cell lines

Treated samples	Inhibitory effect on cell lines (IC_50_)				
	Vero (60 µg mL^−1^) (%)	MDA-MB-231 (20 µg mL^−1^) (%)	HeLa (20 µg mL^−1^) (%)	SiHa (20 µg mL^−1^) (%)	Hep-G2 (30 µg mL^−1^) (%)
Cu-MWCNTs	53.54	58.89	55.77	56.78	51.55
Ag-MWCNTs	52.04	58.56	52.45	58.54	50.66

Each value represents mean ± SD

## Conclusion

A facile and biocompatible route is developed here for the functionalization of CuNPs and AgNPs with MWCNTs. These biohybrid nanomaterials, Cu-MWCNTs and Ag-MWCNTs, have been characterized in detail, and their antimicrobial and anticancer activities are tested. The formation of CuNPs or AgNPs and their functionalization with MWCNTs via bioactive molecules of plant (*T. arjuna*) extract were evident from UV–Vis. spectra, XRD, FTIR, FESEM, EDX, and TEM images. These phytochemically functionalized biohybrid nanomaterials have shown enhanced biocide activity against *E. coli*, *S. aureus, S. typhi*, and *P. aeruginosa*. Furthermore, they are found to be non-toxic to normal epithelial cells (Vero), however, tested highly toxic for human cancer cells. The cell viability was found to decrease with the increasing dose, 10–50 µg mL^−1^, as well as incubation time, 24–72 h. At lower dose of 10 µg mL^−1^, cell viability was found to be ~91 % for normal Vero cells and ~76 % for cancer cells. At 50 µg mL^−1^, the cell viability of normal cells was ~60 % and cancer cells viz., MDA-MB-231, HeLa, SiHa, and Hep-G2 was were dropped to 12, 15, 13, and 20 %, respectively. In summary, the new strategy to obtain the biohybrid nanomaterials seemed to be highly beneficial especially for biomedical applications.
